# 

**Published:** 1866-11

**Authors:** 


### CONTENTS.

ORIGINAL COMMUNICATIONS.

Professional Longings ........ 471
Vital Function in Dentine ........ 477
The Use of the Mallet ........ 483
Exposed Pulps ........ 487
Ten years in the Profession ........ 495
The Rubber Company and the American Dental Association ........ 501

PROCEEDINGS OF SOCIETIES.

Ohio State Dental Society ........ 502

EDITORIAL.

The Rubber Question ........ 513
Personal and Selfish ........ 515
Display of Titles ........ 516
A Useless Dispute settled ........ 517
The Ohio Dentists and the Goodyear Dental Vulcanite Company ........ 518
Massachusetts Dental Society ........ 518

